# Baldness

**Published:** 1883-07

**Authors:** 


BALDNESS.

In an article recently contributed to the *Gesundheit*—a paper, as its name imports, devoted to sanitary subjects—Professor Reclam, a German *Gelehrter*, makes some timely and useful observations on the subject of baldness. After describing, in a vein of pleasantry, the vast array of bare polls which may be seen any evening in the pit of a theater or the body of a lecture-room, he discusses the causes of baldness. He does not think, as is sometimes said, that loss of hair is the result either of impaired health or of much study. The strongest men are often bareheaded, and German professors, who are nothing if not studious, are distinguished above all men by the profusion of their locks. On the other hand, soldiers and postilions, who wear heavy helmets and leather caps, and wear them a good deal, are frequently as bald as billiard balls. From these facts Herr Reclam draws the conclusion that baldness comes chiefly of the artificial determination of blood to the head, and to the heat and perspiration thence arising. The result is a relaxed condition of the scalp and loss of hair. If the skin of the head be kept in a healthy state, contends the professor, the hair will not fall off. To keep it healthy, the head-covering should be light and porous, the head kept clean by washings with water, and the hair cut short. The nostrums vended as hair restorers, and on which a fabulous amount of money is wasted by the ignorant for the benefit of quacks, he denounces as worse than useless. In ninety-nine cases out of a hundred they are worse than useless. Cleanliness and cold water are the sole trustworthy specifics; but when once the hair roots are destroyed, not all the oil of Macassar, the bear's grease of Siberia, nor the cantharides of Spain will woo back the vanished locks.—*Scientific American.*

